# The OPERA trial - comparison of early nasal high flow oxygen therapy with standard care for prevention of postoperative hypoxemia after abdominal surgery: study protocol for a multicenter randomized controlled trial

**DOI:** 10.1186/1745-6215-14-341

**Published:** 2013-10-18

**Authors:** Emmanuel Futier, Catherine Paugam-Burtz, Jean-Michel Constantin, Bruno Pereira, Samir Jaber

**Affiliations:** 1 Department of Anesthesiology and Critical Care Medicine, Estaing Hospital, University Teaching Hospital of Clermont-Ferrand, 1, place Lucie Aubrac, Clermont-Ferrand Cedex 1, 63000, France; 2 Department of Anesthesiology and Critical Care Medicine B (DAR B), Saint-Eloi Hospital, University Teaching Hospital of Montpellier, 80 avenue Augustin Fliche, Montpellier, 34295, France; 3 Department of Anesthesiology and Critical Care Medicine, AP-HP Beaujon Hospital, 100, boulevard du Général Leclerc, Clichy, 92118, France; 4 Biostatistics Unit, Direction de la Recherche Clinique (DRCI), 58 rue Montalembert, Clermont-Ferrand, 63003, France

**Keywords:** Postoperative hypoxemia, Oxygen therapy, Postoperative pulmonary complications, Abdominal surgery, Noninvasive respiratory support

## Abstract

**Background:**

Respiratory support following postoperative extubation is of major importance to prevent hypoxemia and subsequent respiratory failure and reintubation. High-flow nasal cannula oxygen (HFNC) delivers a flow-dependent positive airway pressure and improves oxygenation by increasing end-expiratory lung volume. Whether application of HFNC may have therapeutic advantages over conventional oxygen therapy for respiratory support in the early postextubation surgical period remains to be established.

**Methods/design:**

The Optiflow for prevention of post-extubation hypoxemia after abdominal surgery (OPERA) trial is an investigator-initiated multicenter randomized controlled two-arm trial with assessor-blinded outcome assessment, randomizing 220 patients with intermediate to high risk of pulmonary complications after abdominal surgery to receive HFNC or conventional oxygen therapy following extubation, stratified by the presence of epidural analgesia and center. The primary outcome measure is the percentage of patients with postoperative hypoxemia one hour after tracheal extubation. Secondary outcome measures are postoperative pulmonary complications, need for noninvasive ventilation and intubation for respiratory failure.

**Discussion:**

The OPERA trial is the first randomized controlled study powered to investigate whether early application of HFNC following extubation after abdominal surgery prevents against postoperative hypoxemia and pulmonary complications.

**Trial registration:**

ClinicalTrials.gov Identifier: NCT01887015.

## Background

Recent data suggest that more than 200 million major surgical procedures are performed worldwide each year [1]. Postoperative respiratory complications still remain a frequent problem, thereby increasing postoperative morbidity and mortality, [2] and putting a burden on hospital length of stay and costs of health care [3].

Respiratory support is of major importance following extubation to avoid respiratory failure and reintubation. It has been reported that postoperative hypoxemia complicates between 30% and 50% of cases after abdominal surgery, even among those undergoing uneventful procedures [4,5]. After extubation and with spontaneous breathing at ambient air, derecruitment of lung areas and loss of functional alveolar units may rapidly occur leading to hypoxemia [6]. Supplemental oxygen administration, usually by the use of nasal prongs or a facemask, is required to ensure adequate arterial oxygenation after extubation [7]. However, although oxygen therapy is effective in treating most cases of postoperative hypoxemia [8], low ventilation-perfusion ratios may be only partially responsive to an increase in oxygen concentration [9]. In addition, oxygen therapy is only a symptomatic approach and is ineffective to compensate for loss in lung volume. In contrast, the noninvasive application of continuous positive airway pressure is effective to maintain lung volume and to improve gas exchange after major surgery [10]. A randomized controlled study authored by Squadrone and colleagues [11] demonstrated that application of continuous positive airway pressure (CPAP) may reduce the need for reintubation, pneumonia and postoperative sepsis in patients who developed acute hypoxemia (defined as an arterial oxygen tension to inspiratory oxygen fraction ratio (PaO_2_/FiO_2_) of 300 mmHg or less) after elective major abdominal surgery. However, patient cooperation and tolerance, air leaks and gastric distension are crucial for application and success of CPAP or noninvasive ventilation (NIV). Moreover, NIV affects health-care utilization, since its application usually requires admission to intensive care or within care structures capable of providing high levels of monitoring.

High-flow nasal cannula (HFNC), which delivers high-flow heated and humidified oxygen and air via nasal prongs at a prescribed fraction of inspired oxygen and a maximum flow of 60 L/min, is an attractive alternative to conventional oxygen therapy [12,13]. Previous studies have shown that HNFC oxygen therapy generates a flow-dependent positive airway pressure [13,14] and improves oxygenation by increasing end-expiratory lung volume, thus suggesting a possible alveolar recruitment associated with high-flow therapy [15]. Although prophylactic use of HFNC may have potential therapeutic advantages over conventional oxygen therapy for respiratory support after extubation, evaluation is limited in the early post-extubation surgical period and benefit remains to be established.

The OPERA study aims to compare the effects of early application of HFNC oxygen therapy and conventional oxygen administration after extubation on postoperative pulmonary complications in patients with intermediate to high risk of postoperative pulmonary complications after abdominal surgery.

## Methods/design

### Objectives and design

The Optiflow for prevention of post-extubation hypoxemia after abdominal surgery (OPERA) study is an investigator-initiated multicenter randomized controlled two-arm trial. The Institutional Review Board of the University Hospital of Clermont-Ferrand (France) approved the trial. By April 25 2013, the study had been approved by all centers by a central ethics committee (Comité de Protection des Personnes Sud-Est VI, Clermont-Ferrand, France) with the registration number IDRCB 2013-A00030-45. The OPERA study is conducted in accordance with the declaration of Helsinki and was registered on June 24 2013 at http://www.clinicaltrials.gov with trial identification number NCT01887015.

### CONSORT diagram

**Figure 1:**
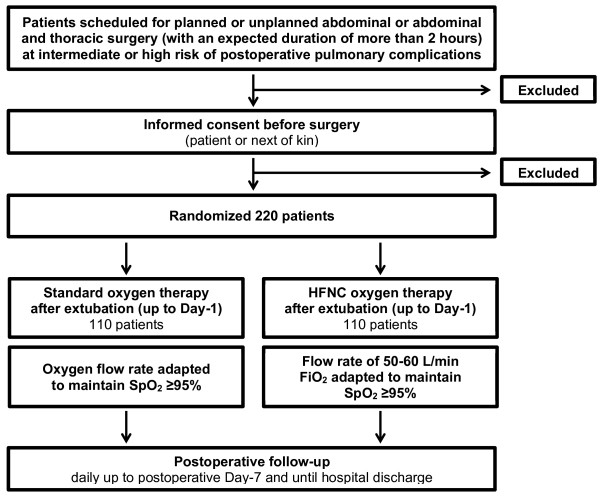
CONSORT diagram of the OPERA trial.

Figure 1 shows the CONSORT diagram of the OPERA trial.

### Study population

Local investigators screen consecutive patients scheduled for planned or unplanned abdominal or abdominal and thoracic surgery with an expected duration of two hours or more in participating centers. Patients with intermediate to high risk for postoperative pulmonary complications following abdominal surgery with general anesthesia are eligible for participation. To identify such patients, the ARISCAT risk score will be used [16]. An ARISCAT risk score ≥26 is associated with an intermediate to high risk for postoperative pulmonary complications.

Patients fulfilling one or more of the following criteria will not be included: lack of informed consent prior to randomization (for an unplanned surgical procedure consent is obtained from the next of kin), nonadult patients (age <18 years), patients with a body mass index ≥35 kg/m^2^, a life-threatening condition requiring emergency surgery, patients with obstructive sleep apnea, and pregnant patients to provide a relatively homogenous study population and avoid potential confounding in the interpretation.

All patients are asked for signed informed consent, as required by the institutional review board in accordance with the Declaration of Helsinki.

### Randomization

Randomization is performed using a dedicated centralized telephone system, accessible round the clock, and using a computer-generated allocation sequence. The randomization sequence is generated using random blocks and is stratified per center and according to the planned use or nonuse of postoperative epidural analgesia, which is a factor that may influence outcomes [17]. No blocking is applied to other trial factors.

### Trial interventions

Patients eligible for inclusion following extubation after abdominal surgery are randomly assigned to standard oxygen therapy via either nasal prongs or face mask with oxygen flow titrated by the bedside clinician to maintain a peripheral oxygen saturation of 95% or more, or HFNC oxygen therapy at a flow rate of 50 to 60 L/min and FiO_2_ titrated by the bedside clinician to maintain a peripheral oxygen saturation of 95% or more. In each group, extubation criteria after surgery are: recovery of spontaneous ventilation with an expired tidal volume between 5 and 8 ml/kg, respiratory rate between 12 and 25 breaths/min, absence of residual neuromuscular blockade (assessed by a T4/T1 ratio ≥90%), peripheral oxygen saturation ≥95%, stable hemodynamics and a body temperature ≥36°C. HFNC oxygen therapy is delivered via the Optiflow™ system (Fisher & Paykel Healthcare Ltd, Auckland, New Zealand) using a MR850 heated humidifier and a RT202 breathing circuit. Allocated therapy is delivered until 0700 to 0800 hours postoperative day 1 at which time point study therapy is discontinued and requirement for supplemental oxygen therapy is assessed.

### Standard procedures

The recently completed Intraoperative Protective Ventilation (IMPROVE) trial showed significantly improved clinical outcomes (10.5% versus 27.5%, *P* <0.001) in patients who received intraoperative lung-protective mechanical ventilation compared with those receiving NIV [18]. Therefore, the study protocol stresses that intraoperative lung-protective mechanical ventilation must be used in the OPERA trial.

All other aspects of patient care during the intra- and postoperative periods, including general anesthesia, fluid administration, prophylactic antibiotics and postoperative pain management, are conducted by the attending physician according to the expertise of the staff at each center and routine clinical practice. However, it is suggested to perform postoperative pain management to achieve a visual numerical scale (VNS) pain score <3, to avoid excessive administration of intraoperative fluids (according to the discretion of the attending anesthesiologist), and to use appropriate prophylactic antibiotics. Data on the applied procedures will be collected and analyzed.

### Study endpoints

Primary outcome variable is the percentage of patients who developed postoperative hypoxemia one hour after tracheal extubation. Postoperative hypoxemia is defined as PaO_2_/FiO_2_ ratio <300, as used previously [11].

Secondary outcome variables are postoperative gas exchange one hour after extubation and after discontinuation of the allocated treatment, respiratory discomfort (using a numerical rating scale, ranging from 0 to 10), need for supplemental oxygen therapy (defined as arterial oxygen saturation <93% on room air), need for postoperative reintubation and/or NIV for acute respiratory failure within the first seven days after surgery, postoperative pulmonary complications within the first seven days after surgery, unexpected intensive care unit (ICU) admission or readmission, ICU and hospital length of stays, and in-hospital mortality. Acute respiratory failure is defined by one of the hypoxemic criteria (peripheral oxygen saturation (SpO_2_) <92% while breathing at least 10 L/min oxygen, PaO_2_ <60 mmHg on air or PaO_2_ <80 mmHg while breathing any supplemental oxygen) and at least one of the following criteria: severe respiratory distress with dyspnea, accessory muscle recruitment and paradoxical abdominal or thoracic motion, respiratory rate >25 breaths/min, respiratory acidosis with pH <7.30 and arterial carbon dioxide partial pressure (PaCO_2_) >50 mmHg [19]. Postoperative pulmonary complications are scored with the use of a graded scale [20] from 0 (no pulmonary complications) to 4 (the most severe complications). Postoperative pulmonary complications are defined as: atelectasis (opacification of the lung with shift of the mediastinum, hilum or hemidiaphragm toward the affected area and compensatory overinflation in the adjacent nonatelectatic lung); suspected pulmonary infection (patient receives antibiotics and meets at least one of the following criteria: new or changed sputum, new or changed lung opacities on chest X-ray when clinically indicated, tympanic temperature >38.3°C, white blood cell (WBC) count >12,000/μl in the absence of other infectious focus); pleural effusion (chest X-ray demonstrating blunting of the costophrenic angle, loss of the sharp silhouette of the ipsilateral hemidiaphragm in upright position, evidence of displacement of adjacent anatomical structures or (in supine position) a hazy opacity in one hemithorax with preserved vascular shadows); and pneumothorax (air in the pleural space with no vascular bed surrounding the visceral pleura).

### Blinding

A computer program will generate the coding list and will allocate coding numbers to patients from the specific trial site. It is not feasible to mask the assigned oxygen therapy from the attending anesthesiologists, since they have an ethical responsibility to ensure patient safety until discharge from the post-anesthesia care unit. However, in each participating center, postoperative data will be collected and recorded onto case report forms (CRFs) by local research coordinators blinded to the randomized intervention. A trained research coordinator, blinded to the randomized intervention, will centralize data from all sites and will record them onto the electronic database.

### Suspension of protocol

The protocol may be suspended for the individual patient at the discretion of the attending anesthesiologist if the patient is to be intubated in the presence of acute respiratory failure. In case of severe respiratory discomfort with the assigned therapy, the protocol may be temporarily suspended. The protocol will be resumed promptly after resolution of symptoms. These patients will be analyzed according to their originally assigned groups on an intention-to-treat basis.

### Statistics

For this study, 2 × 110 patients are needed to detect a 50% relative difference in the primary outcome, at a two-sided α level of 0.05 and a statistical power of 90%, assuming a 40% rate of postoperative hypoxemia in the standard oxygen therapy group [4,5]. An interim analysis is planned after enrollment of the first 110 patients to review data relating to patient safety and quality of trial conduct using the Lan and DeMets method (East software, Cytel Inc., Cambridge, MA, USA). The data and monitoring safety committee will recommend stopping the trial if the continued conduct of the trial compromises patient safety (a between-group difference in serious adverse events is found).

Statistical analysis will be conducted on an intention-to-treat (ITT) basis. Unadjusted chi-square or Fisher’s exact test will be used for primary outcome analysis. Adjusted analysis will be performed with the use of robust random effects Poisson generalized linear regression (1) to take into account adjustment on possible confounding covariates selected according to clinical relevance and (2) to consider within and between center variability. All effect sizes will be presented with 95% confidence intervals. The chi-square test (or Fisher’s exact test as appropriate) will be used for secondary binary outcomes. Continuous variables will be compared with the use of the unpaired *t* test or the Mann–Whitney *U* test when appropriate. The Shapiro-Wilk test will be used to assess normality, and the Fisher-Snedecor test to assess homoscedasticity. Adjusted analyses will be performed using the same adjustment variables, described previously, in the linear regression model. The time-to-event curves should be calculated with the use of the Kaplan-Meier method. If the frequency of missing data is >5%, an additional analysis will be performed using the multiple imputation method (STATA command mi) [21]. Longitudinal analysis using mixed models will be used to take into account between and within subject variability. All analyses will be conducted with Stata statistical software, version 12 (StataCorp LP, College Station, TX, USA). A two-sided *P* value of less than 0.05 will be considered to indicate statistical significance.

### Registration

Data will be collected and recorded onto CRFs in each center by trained local research coordinators blinded to the randomized intervention. A trained research coordinator will centralize data from all sites and will record them onto the electronic database.

### The following data will be collected and registered

Prerandomization and baseline characteristics: gender, age, height, weight, ideal body weight, physical status (according to the American Society of Anesthesiology status score), cardiac status (hypertension, cardiac failure, ischemic heart disease), respiratory status (history of chronic obstructive pulmonary disease, asthma status, respiratory infection in the last month, long-term home oxygen therapy), smoking status, alcohol status, history of diabetes mellitus, recent denutrition (defined as weight loss >10% in the last six months and/or plasma albumin <30 g/l), history of renal insufficiency, ARISCAT score, preoperative SpO_2_, and blood samples (WBC count, hematocrit, hemoglobin, platelet count, prothrombin time, fibrinogen, urea, creatinine, C reactive protein and albumin).

During the surgical and anesthesia procedures: type of surgery (planned or unplanned), surgical site (abdominal or abdominal and thoracic), surgical technique (laparotomy or laparoscopy), duration of both anesthesia and surgical procedures, blood losses, transfusion requirement, all administered drugs during anesthesia (anesthetics, vasoactive drugs, neuromuscular blocking agent), all administered fluid (crystalloid, colloid), ventilator settings (tidal volume, positive end-expiratory pressure, recruitment maneuver, FiO_2_, plateau and peak pressures), duration of mechanical ventilation.

From randomization to postoperative day 1 (at which time point study therapy is discontinued): vital parameters, SpO_2_, visual analog scale (VAS) score for pain, VNS score for respiratory discomfort, arterial blood gases (PaO_2_, PaCO_2_, pH, bicarbonate test (HCO_3_), arterial oxygen saturation (SaO_2_)), all side effects related to the allocated oxygen therapy, presence or absence of postoperative pulmonary complications.

Daily from postoperative day 1 to day 7: vital parameters, need for supplemental oxygen administration, presence or absence of postoperative pulmonary complications (pulmonary complication, if present, is graded), chest-X ray when clinically indicated, need for intubation or noninvasive mechanical ventilation, need for ICU admission or readmission. The day of development of any pulmonary complication and/or ICU admission is indicated.

The day of hospital discharge: duration of ICU and hospital stays, survival status (the day of death, if present, is indicated).

### Data handling and record keeping

Data will be handled according to French law. All original records (including consent forms, CRFs and relevant correspondence) will be archived at trial sites for 15 years. The clean database file will be anonymized and maintained for 15 years.

### Study organization

The study is an investigator-initiated trial. Study promotion is performed by Clermont-Ferrand University Hospital, Clermont-Ferrand, France. There is no industry support or involvement in the trial.

### Duration and timeline

Patients from three French University Teaching Hospitals are expected to be included during a two-year inclusion period starting September 2013.

2013: Protocol, approvals from ethics committee, and trial tool development (CRF, randomization system).

2013 to 2014: Inclusion of patients.

2014: Cleaning and closure of the database. Data analyses and writing of the manuscript, and submission for publication.

## Discussion

It has become clear that postoperative pulmonary complications are the main source of postoperative morbidity and mortality [2,22]. Optimizing respiratory support is, therefore, of particular importance during the perioperative period, especially in patients with high risk of pulmonary complications. The OPERA trial is the first randomized controlled study powered to investigate early application of HFNC oxygen therapy in patients scheduled for abdominal surgery with intermediate to high risk of pulmonary complications.

The primary endpoint of the trial is postoperative hypoxemia, which could be seen as a shortcoming since correction of hypoxemia is only purely symptomatic and does not always improves postoperative outcomes of surgical patients. The incidence of postoperative hypoxemia is particularly high in surgical patients, reaching up to 50% [4,5]. The ability to anticipate and to provide early treatment to potentially modifiable adverse clinical events such as postoperative hypoxemia is of critical importance to prevent the development of subsequent complications. Moreover, since we collect and report on most postoperative pulmonary complications, it may still be possible to determine the effects on other complications.

The selection of patients who may benefit from respiratory support therapy in the postoperative period is of particular importance to design individually tailored management approaches. In this study, we use the ARISCAT score [16] to assess the individual predictive risk of postoperative pulmonary complications, and the focus is on investigating patients with intermediate to high risk of complications. The ARISCAT score includes seven independent risk factors (four patient-related risk factors and three related to the surgical procedure, accounting for 55% and 45% of the score respectively), with the most relevant cut point being the risk score of 26.

Several confounding factors, such as postoperative pain management, intraoperative fluid administration and respiratory chest physiotherapy, can be suggested. These points are not protocolized in the OPERA trial, in contrast to the management of intraoperative mechanical ventilation. Nevertheless, the study protocol stresses that intra- and postoperative care are to be performed in accordance with each center’s specific expertise to make the trial as close as possible to routine clinical care.

In conclusion, the OPERA trial is an investigator-initiated pragmatic randomized controlled trial powered to test the hypothesis that early application of HFNC prevents postoperative hypoxemia in patients with intermediate to high risk of postoperative pulmonary complications. The OPERA trial also determines the effects of high-flow oxygen therapy on most of the postoperative pulmonary complications, need for noninvasive mechanical ventilation or intubation for postoperative respiratory failure, and lengths of ICU and hospital stays.

## Trial status

The trial is ongoing and is actively enrolling.

## Abbreviations

CPAP: Continuous positive airway pressure; CRF: Case report form; HCO3: Bicarbonate test; HFNC: High-flow nasal cannula oxygen therapy; NIV: Noninvasive ventilation; PaCO2: Arterial carbon dioxide partial pressure; PaO2/FiO2: Arterial oxygen tension to inspiratory oxygen fraction ratio; SaO2: Arterial oxygen saturation; SpO2: Peripheral oxygen saturation; VAS: Visual analog scale; VNS: Visual numerical scale; WBC: White blood cell.

## Competing interests

SJ received grants from Fisher & Paykel France for consulting. The other authors declare that they have no competing interests.

## Authors’ contributions

EF drafted the manuscript together with SJ and CPB. EF designed the study together with SJ, CPB and JMC. BP planned the statistical analysis. All authors read and approved the final manuscript.

## References

[B1] Weiser TG Regenbogen SE Thompson KD Haynes AB Lipsitz SR Berry WR Gawande AA An estimation of the global volume of surgery: a modelling strategy based on available data Lancet 2008 372 139 144 10.1016/S0140-6736(08)60878-8 18582931

[B2] Khuri SF Henderson WG DePalma RG Mosca C Healey NA Kumbhani DJ Determinants of long-term survival after major surgery and the adverse effect of postoperative complications Ann Surg 2005 242 326 341 16135919 10.1097/01.sla.0000179621.33268.83 PMC1357741

[B3] Shander A Fleisher LA Barie PS Bigatello LM Sladen RN Watson CB Clinical and economic burden of postoperative pulmonary complications: patient safety summit on definition, risk-reducing interventions, and preventive strategies Crit Care Med 2011 39 2163 2172 10.1097/CCM.0b013e31821f0522 21572323

[B4] Arozullah AM Daley J Henderson WG Khuri SF Multifactorial risk index for predicting postoperative respiratory failure in men after major noncardiac surgery. The national veterans administration surgical quality improvement program Ann Surg 2000 232 242 253 10.1097/00000658-200008000-00015 10903604 PMC1421137

[B5] Lawrence VA Dhanda R Hilsenbeck SG Page CP Risk of pulmonary complications after elective abdominal surgery Chest 1996 110 744 750 10.1378/chest.110.3.744 8797421

[B6] Lindberg P Gunnarsson L Tokics L Secher E Lundquist H Brismar B Hedenstierna G Atelectasis and lung function in the postoperative period Acta Anaesthesiol Scand 1992 36 546 553 10.1111/j.1399-6576.1992.tb03516.x 1514340

[B7] Rosenberg J Oturai P Erichsen CJ Pedersen MH Kehlet H Effect of general anesthesia and major versus minor surgery on late postoperative episodic and constant hypoxemia J Clin Anesth 1994 6 212 216 10.1016/0952-8180(94)90061-2 8060628

[B8] Fu ES Downs JB Schweiger JW Miguel RV Smith RA Supplemental oxygen impairs detection of hypoventilation by pulse oximetry Chest 2004 126 1552 1558 10.1378/chest.126.5.1552 15539726

[B9] Whiteley JP Gavaghan DJ Hahn CE Variation of venous admixture, SF6 shunt, PaO2, and the PaO2/FIO2 ratio with FIO2 Br J Anaesth 2002 88 771 778 10.1093/bja/88.6.771 12173192

[B10] Jaber S Chanques G Jung B Postoperative noninvasive ventilation Anesthesiology 2010 112 453 461 10.1097/ALN.0b013e3181c5e5f2 20068454

[B11] Squadrone V Coha M Cerutti E Schellino MM Biolino P Occella P Belloni G Vilianis G Fiore G Cavallo F Ranieri VM Continuous positive airway pressure for treatment of postoperative hypoxemia: a randomized controlled trial JAMA 2005 293 589 595 10.1001/jama.293.5.589 15687314

[B12] Chanques G Constantin JM Sauter M Jung B Sebbane M Verzilli D Lefrant JY Jaber S Discomfort associated with underhumidified high-flow oxygen therapy in critically ill patients Intensive Care Med 2009 35 996 1003 10.1007/s00134-009-1456-x 19294365

[B13] Chanques G Riboulet F Molinari N Carr J Jung B Prades A Gallia F Futier E Constantin JM Jaber S Comparison of three high flow oxygen therapy delivery devices: a clinical physiological cross-over study Minerva Anestesiol 2013 Epub ahead of print 23857440

[B14] Parke R McGuinness S Eccleston M Nasal high-flow therapy delivers low level positive airway pressure Br J Anaesth 2009 103 886 890 10.1093/bja/aep280 19846404 PMC2777940

[B15] Corley A Caruana LR Barnett AG Tronstad O Fraser JF Oxygen delivery through high-flow nasal cannulae increase end-expiratory lung volume and reduce respiratory rate in post-cardiac surgical patients Br J Anaesth 2011 107 998 1004 10.1093/bja/aer265 21908497

[B16] Canet J Gallart L Gomar C Paluzie G Valles J Castillo J Sabate S Mazo V Briones Z Sanchis J Prediction of postoperative pulmonary complications in a population-based surgical cohort Anesthesiology 2010 113 1338 1350 10.1097/ALN.0b013e3181fc6e0a 21045639

[B17] Popping DM Elia N Marret E Remy C Tramer MR Protective effects of epidural analgesia on pulmonary complications after abdominal and thoracic surgery: a meta-analysis Arch Surg 2008 143 990 999 10.1001/archsurg.143.10.990 18936379

[B18] Futier E Constantin JM Paugam-Burtz C Pascal J Eurin M Neuschwander A Marret E Beaussier M Gutton C Lefrant JY Allaouchiche B Verzilli D Leone M De Jong A Bazin JE Pereira B Jaber S A trial of intraoperative low tidal volume in abdominal surgery N Engl J Med 2013 369 428 437 10.1056/NEJMoa1301082 23902482

[B19] Jaber S Delay JM Chanques G Sebbane M Jacquet E Souche B Perrigault PF Eledjam JJ Outcomes of patients with acute respiratory failure after abdominal surgery treated with noninvasive positive pressure ventilation Chest 2005 128 2688 2695 10.1378/chest.128.4.2688 16236943

[B20] Hulzebos EH Helders PJ Favie NJ De Bie RA de la Brutel RA Van Meeteren NL Preoperative intensive inspiratory muscle training to prevent postoperative pulmonary complications in high-risk patients undergoing CABG surgery: a randomized clinical trial JAMA 2006 296 1851 1857 10.1001/jama.296.15.1851 17047215

[B21] Royston P Multiple imputation of missing values Stata J 2004 4 227 241

[B22] Thompson JS Baxter BT Allison JG Johnson FE Lee KK Park WY Temporal patterns of postoperative complications Arch Surg 2003 138 596 602 10.1001/archsurg.138.6.596 12799329

